# Food consumption habits in two states of Australia, as measured by a Food Frequency Questionnaire

**DOI:** 10.1186/1756-0500-4-507

**Published:** 2011-11-23

**Authors:** Alison M Daly, Jacqueline E Parsons, Nerissa A Wood, Tiffany K Gill, Anne W Taylor

**Affiliations:** 1 Health Outcomes Assessment, Epidemiology Branch, Public Health Intelligence, Public Health Division, Western Australian Department of Health. 189 Royal Street, East Perth, Western Australia, Australia 6004; 2 Population Research and Outcomes Studies, University of Adelaide, South Australia, Australia. 122 Frome Street, Adelaide SA 5000

## Abstract

**Background:**

Obesity is an important public health problem in Australia, and monitoring the nutritional intake of the population is an important endeavour. One way to assess food habits is via Food Frequency Questionnaires (FFQ). This pilot study used a routine telephone risk factor surveillance survey to recruit participants in South Australia (SA) and Western Australia (WA) to a postal survey investigating food consumption habits, using a FFQ. Respondents were also asked specific additional questions about their fruit and vegetable consumption and also about their height and weight so that comparisons could be made between the data collected in the risk factor surveillance system and the postal survey.

**Findings:**

In total, 1275 respondents (65% of eligible telephone respondents) completed the postal survey. The results of the FFQ were very similar for WA and SA. Western Australians consumed statistically significantly more serves of vegetables than South Australians (t = 2.69 df = 1245 p <= 0.01), and females consumed statistically significantly more serves of both fruit and vegetables than males (t = 4.51 df = 1249 p <= 0.01 and t = 4.83 df = 1249 p <= 0.01 respectively). Less than 10% of respondents met the daily guidelines for vegetable consumption. Over half of respondents were overweight or obese.

**Conclusions:**

Although a wide variety of foods were consumed, guidelines for fruit and vegetable consumption were not being met and overweight and obesity continue to be issues in this population.

## Background

Obesity is an important health issue for Australia, recognised as a National Health Priority Area by the Australian Health Ministers Advisory Council [1]. The prevalence of overweight and obesity has been growing over the past three decades, with latest national self-reported figures estimating half of Australian adults to be overweight or obese [2]. Healthy weight and good nutrition are the focus of health promotion programs at the federal, state and local level [3-5]. Evaluation of health promotion interventions in regard to nutritional intakes and monitoring of trends in weight in the population are therefore essential components of Australia's attempts to bring overweight and obesity under control.

Ongoing risk factor surveillance, like that undertaken by South Australia (SA) and Western Australia (WA) using computer-assisted telephone interviews (CATI), is an ideal vehicle for monitoring health priorities such as obesity [6,7]. Surveillance is useful in itself for monitoring health indicators, but does not usually permit in-depth or lengthy investigation [8]. When more detailed examination of a particular health topic is warranted, surveillance systems, by their very nature, do not permit this level of additional investigation and other methodologies such as postal surveys, face to face interviews, internet surveys or participation in biomedical tests or physical examinations are often used. These alternative methods can enable more complex or lengthy questions to be asked, and can help respondents with visual aids [9]. Techniques such as these may be useful when topics like food consumption are investigated. Respondents can take their time with their responses, which in some circumstances may add to the survey validity [10]. Ongoing surveillance systems can provide a ready-made and easily accessible resource for further investigations, as respondents can be asked during the surveillance survey interview if they would be prepared to participate in additional research. This not only provides an instant sample for the next stage of the research, but it enables comparisons between the different methods if some of the survey content is repeated in the second stage.

Measuring people's food consumption and nutritional intake is difficult, and usually relies on self-report. This has some limitations, such as giving 'socially desirable' responses, under or over-estimating the foods consumed, or recall bias [10]. It is also limited by the time available to investigate consumption of the many different types of food that are of interest. Food frequency questionnaires (FFQ) are one of the most common approaches to measuring nutritional intake, and these types of instruments are widely used [11]. To examine the eating habits of Australian adults, a pilot study of mixed-mode research was undertaken in WA and SA. Respondents to routine surveillance surveys were recruited to participate in a postal survey about food consumption. A previous paper has reported on key comparisons and addressed some of the methodological issues [12]. This paper aims to assess the frequency of the consumption of various food groups using the combined data from WA and SA.

The South Australian Monitoring and Surveillance System (SAMSS) and the West Australian Health and Wellbeing Surveillance System (HWSS) are ongoing monthly surveys of a random sample of South and Western Australians, respectively [7]. The surveys use Computer Assisted Telephone Interviewing (CATI). Households with a number listed in the Electronic White Pages are eligible for selection. One person in each randomly selected household is selected for interview, and there is no replacement for non-contactable persons.

In the SAMSS and HWSS surveys of October and November of 2006, respondents 18 years of age and over were asked if they would be willing to complete the FFQ. The FFQ was based on the Australian Health Measurement Survey conducted by the Australian Institute of Health and Welfare in 2004, with some minor variations [13]. The FFQ was mailed to respondents who agreed to participate, and they were also asked for permission to link the data in the FFQ to the data collected in the original CATI survey.

The FFQ asked respondents to indicate how often they consumed different foods, with the eight possible response categories ranging from never to four or more times per day. The eight broad food groups and the number of specific foods covered were dairy foods (9); bread and cereal foods (9); meat, fish and egg (20); vegetables (26); fruit (10); baked goods and snacks (12); sugar, spread and dressings (7); and, non-milk beverages (15). Four additional questions were included that were identical to those asked in the surveillance systems. These were how many serves of fruit and vegetables they usually consumed (with a definition of what constitutes a serve provided) and their weight and height. The FFQ also included questions about age (recoded into 18-44, 45-64 and 65+ years), sex and area of residence.

As the data were representing two different populations with two different age and sex structures, the data from each state were weighted by the appropriate state data accommodating their individual sampling strategy. The FFQ data were weighted to compensate for the over-sampling in the rural and remotes areas of WA and in rural areas of SA and then adjusted to the 2005 Estimated Resident Population (ERP) for each state, released by the ABS in June 2006 [14,15].

## Findings

In SA, 1061 respondents were asked to participate in the FFQ, with 832 (84.2%) agreeing to participate and 634 (59.8%) completing the survey. In WA, 901 respondents were asked to participate in the FFQ, with 735 (84.9%) agreeing to participate and 641 (71%) completing the survey.

The description of the study population is shown in Table 1 with the weighted samples from the two States similar in their demographic composition.

**Table 1:** Demographic characteristics of all respondents to the FFQ

	SA		WA		TOTAL	
	**n**	%	**n**	%	**n**	%

**Sex**						

Male	311	49.1	321	50.1	633	49.6

Female	323	50.9	320	49.9	642	50.4

**Age group**						

18 to 44 years	298	47.1	321	50.1	622	48.8

45 to 64 years	208	32.9	208	32.4	416	32.6

65 years +	127	20.1	112	17.4	237	18.6

**Area of residence**						

Metropolitan	462	72.9	501	78.2	968	75.9

Country	172	27.1	140	21.8	307	24.1

**Total**	**634**	**100**	**641**	**100**	**1275**	**100**

In the FFQ, categories for each food were converted into a value equivalent to a daily intake of each food, using the method from the Victorian Cancer Council's FFQ [16]. This figure is equivalent to the number of times per day the food is consumed; however it does not take into account the amount of the food consumed. The conversion is shown in Table 2. The frequency of consumption for each of the individual food items were summed to provide the daily equivalent frequency for each of the broad food groups.

**Table 2:** Daily equivalent frequency

Original frequency	Daily equivalent frequency
Never	0
Less than once per month	0.02
1-3 times per month	0.07
Once per week	0.14
2-4 times per week	0.43
5-6 times per week	0.78
Once per day	1.00
2-3 times per day	2.50
4 or more times per day	4.00

### Frequency of food consumption

The mean daily equivalent frequencies of consuming food in the broad categories described above were calculated for each state, and are presented in Table 3. The results refer to the number of times the foods were consumed, not the quantities. The most striking finding is the degree of similarity between SA and WA for the mean daily equivalent for each food group. Exploration of the expanded food groups showed no significant differences except that participants from SA consumed more mangos and paw paws than those from WA and drank more beer and tea than their WA counterparts. No other differences were statistically significant and most estimates were almost identical.

**Table 3:** Mean daily equivalent frequencies, broad food groups, SA and WA, 2006

Food group	SA		WA	
	**Mean**	**95% CI**	**Mean**	**95% CI**

Dairy	3.6	3.5-3.8	3.5	3.3-3.7

Bread and cereal	2.8	2.8-2.9	2.7	2.5-2.8

Meat, fish and eggs	2.1	2.0-2.2	2.2	2.1-2.4

Vegetables	6.9	6.6-7.2	6.7	6.2-7.2

Fruit	2.3	2.1-2.4	2.4	2.1-2.8

Baked goods and snacks	1.5	1.4-1.6	1.4	1.3-1.6

Sugar, spreads and dressings	2.8	2.7-3.0	2.8	2.6-3.1

Non-milk beverages	7.6	7.4-7.9	7.5	7.1-7.8

In terms of the broad food categories, vegetables and non-milk beverages were the most frequently consumed food group in both states. On average, vegetables were consumed over six times a day and non-milk beverages over seven times a day. Baked goods and snacks are consumed more than twice daily on average, with sugar, spreads and dressings consumed nearly three times a day.

Given the similarity between states, the data were combined and examined by sex and age group. Tables 4 and 5 present the results. Females consumed vegetables and fruit significantly more frequently than males (t = 4.51 df = 1249 p <= 0.01 and t = 4.83 df = 1249 p <= 0.01 respectively). Males consumed more protein rich foods (meat, fish, eggs) (t = 2.09 df = 864 p = 0.04) and more non-milk beverages (t = 2.15 df = 754 p = 0.03) compared with females.

**Table 4:** Mean daily equivalent frequencies by sex, for each broad food category, WA and SA 2006

	Female		Male		Total	
	**mean**	**95% CI**	**mean**	**95% CI**	**mean**	**95% CI**

**Dairy foods**	3.6	3.4-3.7	3.5	3.3-3.8	3.5	3.4-3.7

**Bread and cereal foods**	2.7	2.6-2.8	2.7	2.5-2.9	2.7	2.6-2.8

**Meat, fish, eggs**	2.1	2.0-2.2	2.3	2.1-2.4	2.2	2.1-2.3

**Vegetables**	7.6	7.2-8.0	5.8	5.5-6.2	6.7	6.4-7.0

**Fruit**	2.7	2.5-2.9	1.9	1.7-2.2	2.3	2.2-2.5

**Baked goods and snacks**	1.4	1.3-1.5	1.5	1.4-1.7	1.5	1.4-1.6

**Sugar, spreads and dressings**	2.7	2.5-2.9	2.9	2.7-3.2	2.8	2.6-3.0

**Non-milk beverages**	7.2	7.0-7.4	7.7	7.3-8.1	7.5	7.2-7.7

**Table 5:** Mean daily equivalent frequencies by age group, for each broad food category, WA and SA 2006

	18-44 years		45-64 years		65+years	
	**mean**	**95% CI**	**mean**	**95% CI**	**mean**	**95% CI**

**Dairy foods**	3.6	3.4-3.7	3.5	3.3-3.7	3.3	3.1-3.6

**Bread and cereal foods**	2.7	2.5-2.9	2.6	2.5-2.7	2.9	2.8-3.0

**Meat, fish, eggs**	2.4	2.2-2.6	2.0	1.9-2.1	1.8	1.7-2.0

**Vegetables**	6.8	6.3-7.3	6.7	6.4-7.1	6.6	6.2-6.9

**Fruit**	2.2	1.9-2.5	2.4	2.2-2.5	2.5	2.3-2.7

**Baked goods and snacks**	1.5	1.3-1.6	1.5	1.4-1.6	1.5	1.4-1.7

**Sugar, spreads and dressings**	2.7	2.4-3.0	2.9	2.7-3.0	2.9	2.7-3.1

**Non-milk beverages**	7.5	7.2-8.0	7.5	7.2-7.8	7.0	6.7-7.3

The only significant differences in the mean daily frequency of consumption within food groups by age were: People aged 65 years and over ate from the bread group more often compared with other ages (t = 2.65 df = 874 p <= 0.01); people aged 18-44 years eat from the protein group more often than people aged 45-64 years (t = 3.75 df = 557 p <= 0.01) who eat more often from the protein group than people aged 65 years and over (t = 2.42 df = 800 p = 0.02); and people aged 18 to 64 years drank beverages more often than people aged 65 years and over (t = 2.77 df = 890 p <= 0.01).

### Fruit and vegetable consumption

To specifically assess adherence to healthy eating guidelines, respondents were asked how many serves of vegetables and fruit they consumed each day using the same question wording as used in the surveillance system, with a serve being equivalent to one cup of cooked vegetables or salad, and one cup of diced fruit or one medium or two small pieces of fruit [17]. The mean number of serves of fruit and vegetables consumed by state, sex and age group are presented in Table 6.

**Table 6:** Mean number of serves of fruit and vegetables consumed each day by state, sex and age, WA and SA, 2006

	Vegetables		Fruit	
	**mean**	**95% CI**	**mean**	**95% CI**

**SA**	2.2	2.1-2.3	1.5	1.4-1.6

**WA**	2.5	2.3-2.6	1.6	1.5-1.7

**Male**	2.1	1.9-2.2	1.4	1.3-1.6

**Female**	2.6	2.5-2.7	1.7	1.6-1.8

**18-44**	2.3	2.1-2.4	1.4	1.3-1.6

**45-64**	2.4	2.3-2.5	1.6	1.5-1.8

**65+**	2.5	2.3-2.7	1.8	1.7-1.9

**Persons**	2.4	2.2-2.5	1.6	1.5-1.6

People from WA reported eating significantly more serves of vegetables compared with those from SA (t = 2.69 df = 1245 p <= 0.01). Females reported eating significantly more serves of vegetables (t = 5.19 df = 940 p <= 0.01) and fruit (t = 2.76 df = 809 p <= 0.01) each day compared with males. People aged 18 to 44 years of age reported eating significantly fewer serves of vegetables compared with people aged 65 years and over (t = 2.08 df = 678 p = .04). People aged 18 to 44 years reported eating few serves of fruit each day compared with people aged 45 year and over (t = 3.28 df = 635 p <= 0.01).

The recommended guidelines for vegetable and fruit consumption are five serves of vegetables and two serves of fruit daily. Table 7 shows the proportion of respondents to the FFQ meeting these requirements by state, sex and age group. Less than one in 10 respondents in both states met the guideline for vegetable consumption, with females slightly better than males but little difference by age. Fruit consumption was better, with just under half the respondents eating two or more serves daily; again, a higher proportion of females met the guidelines. The proportion of people meeting the fruit guideline increased with age. There were no significant differences between any groups.

**Table 7:** Proportion of respondents eating at least five serves of vegetables and two serves of fruit per day by state, sex and age group, SA and WA, 2006

	Vegetables		Fruit	
	**%**	**95% CI**	**%**	**95% CI**

**SA**	7.2	5.3-9.7	44.5	39.6-49.6

**WA**	9.0	6.3-12.7	45.6	40.3-51.0

**Male**	6.1	3.5-10.3	37.6	31.7-43.9

**Female**	10.3	8.1-13.0	52.5	48.2-56.7

**18-44**	7.2	4.4-11.6	39.6	33.5-46.2

**45-64**	9.2	6.8-12.3	48.3	43.3-53.3

**65+**	9.2	6.3-13.3	54.5	48.6-60.3

**Persons**	8.2	6.4-10.5	45.2	41.4-48.9

### Body Mass Index

Respondents were asked to give their height without shoes and their weight, and this was converted to body mass index (BMI) using the formula weight in kilograms divided by height in metres squared. The BMI was then classified into three categories: underweight or normal weight (BMI <25), overweight (>= 25 BMI <30) and obese (BMI >= 30) [18]. The mean BMI for both states was within the overweight category, with the mean for SA 26.4 (95% CI 25.9-26.9) and for WA 26.3 (95% CI 25.8-26.8). The results by state, sex and age group are given in Table 8.

**Table 8:** Proportion in BMI categories by state, sex and age group, SA and WA, 2006

	Underweight/ normal weight		Overweight		Obese	
	**%**	**95% CI**	**%**	**95% CI**	**%**	**95% CI**

**SA**	43.6	38.6-48.7	38.8	34.0-43.8	17.6	14.3-21.5

**WA**	46.3	41.0-51.8	38.1	33.0-43.4	15.6	12.3-19.6

**Male**	39.0	32.9-45.4	46.3	40.1-52.5	14.8	11.1-19.5

**Female**	51.3	47.1-55.5	30.6	26.8-34.6	18.1	15.2-21.4

**18-44**	53.0	46.4-59.6	33.9	27.9-40.5	13.0	9.2-18.1

**45-64**	34.5	30.0-39.3	43.0	38.2-48.1	22.4	18.7-26.6

**65+**	43.4	37.8-49.1	41.7	36.0-47.5	15.0	11.6-19.2

**Persons**	45.2	41.4-48.9	38.4	34.8-42.1	16.5	14.0-19.2

## Conclusions

The FFQ showed that South Australians and Western Australians eat some of the foods from each food group daily. The effect of this variety on meeting the recommendations for a healthy diet is hard to assess. The food group list includes foods that are classified as 'healthy' such as fruit and vegetables and are therefore recommended to be eaten frequently. However the food group list also includes foods such as baked goods and snacks and sugary foods where consumption in limited amounts, and less frequently, is recommended. Non-milk beverages, which are consumed the second most frequently of all the food groups, include soft drinks and alcohol which have quite specific guidelines in terms of frequency and amount of consumption. Without examining the frequency patterns for specific foods within each food group, it is difficult to make broad statements about the health impact of frequency of consumption of these groups.

Vegetables, the food group with the highest mean frequency of consumption in the FFQ, were not consumed in sufficient amounts to meet the recommended five serves daily when assessed using the surveillance system definition. Less than one in ten met this guideline even though they were eating from this food group the most often. Fruit consumption was better but less than half of respondents ate the recommended two serves each day [4]. Health promotion programs such as Go for 2 & 5^® ^encourage more fruit and vegetable consumption. It was shown in WA, between 2002 and 2006 that this program increased awareness of the recommended serving of fruit and vegetables and encouraged increased consumption [19]. Even with the increase in consumption, both states still fall short of the recommended guidelines for a healthy diet. The figures presented here are in line with other Australian studies [20].

The continual and ongoing collection of information on these and other important indicators of health increases our capacity to inform both the public and public health policies and interventions of how well Australians are doing and where we need additional efforts. With regard to fruit and vegetables, people, particularly males who are less than 45 years of age, are those who need to be alerted to the substantial shortfall in their consumption of these important food groups.

The FFQ, as originally conceived, does not ask any information about quantity and this is a serious limitation. The Australian Guide to Healthy Eating includes not only recommendations on which foods to eat but also on how much to eat. While the investigation of both quantity and frequency of food consumption is complex and time consuming, it has been shown that including quantity estimates contributes to FFQ validity [21]. More work is needed to find an ideal way to assess these factors on a population basis in a time-efficient, valid and reliable way. The lack of FFQ data on quantity explains the difference between the FFQ mean daily number of serves and the additional data collected assessing the number of serves eaten per day which was based on a tight definition supplied to each respondent. The FFQ lists up to 26 different vegetables and 10 fruits giving the respondent important reminders about fruit and vegetables and perhaps allowing encouragement of a higher estimate of consumption.

In this study, the estimated BMI classified over half of the population as either overweight or obese. Moreover, just over one in six people reported heights and weights that categorised them as obese. This is similar to the proportion of obese adults in Australia as a whole, as measured in the National Health Survey in 2004/5 [22]. Research has shown that people over-report height and under-report weight leading to an underestimation of 'true' BMI in the self-report estimates. This study is therefore likely to have underestimated the prevalence of overweight and obesity in our sample [23]. With health promotion programs addressing obesity likely to become more common, with added investments in terms of time, programs and money, the monitoring and evaluation of the efficacy of these through surveillance systems such as SAMSS and HWSS will become increasingly important.

Overall, this pilot study produced a good response rate, especially for a postal survey, although further work would have to be undertaken to determine if the sub-sample is a representative sample. Notwithstanding, the findings highlight some areas where improvement needs to be made for these populations to reach the healthy eating goals set by the Government. This research has highlighted the value of collaboration and mixed mode methodology in gaining information on the eating habits of Australians.

## Competing interests

The authors declare that they have no competing interests.

## Authors' contributions

AD, TG and AT conceived and designed the study, and oversaw its conduct; TG, AD and NW analysed the data; JP prepared the manuscript; AD, AT and TG revised the manuscript; all authors gave final approval for publication.
